# Inactivation of the *fliY *gene encoding a flagellar motor switch protein attenuates mobility and virulence of *Leptospira interrogans *strain Lai

**DOI:** 10.1186/1471-2180-9-253

**Published:** 2009-12-09

**Authors:** Sumei Liao, Aihua Sun, David M Ojcius, Senlin Wu, Jinfang Zhao, Jie Yan

**Affiliations:** 1 College of Life Sciences, Zhejiang University, Hangzhou 310058, PR China; 2 Department of Medical Microbiology and Parasitology, College of Medicine, Zhejiang University, Hangzhou 310058, PR China; 3 Zhejiang Medical College, Hangzhou 310053, PR China; 4 Health Sciences Research Institute and School of Natural Sciences, University of California, Merced, CA 95343, USA

## Abstract

**Background:**

Pathogenic *Leptospira *species cause leptospirosis, a zoonotic disease of global importance. The spirochete displays active rotative mobility which may contribute to invasion and diffusion of the pathogen in hosts. FliY is a flagellar motor switch protein that controls flagellar motor direction in other microbes, but its role in *Leptospira*, and paricularly in pathogenicity remains unknown.

**Results:**

A suicide plasmid for the *fliY *gene of *Leptospira interrogans *serogroup Icterohaemorrhagiae serovar Lai strain Lai that was disrupted by inserting the ampicillin resistance gene (*bla*) was constructed, and the inactivation of *fliY *gene in a mutant (*fliY*^-^) was confirmed by PCR and Western Blot analysis. The inactivation resulted in the mRNA absence of *fliP *and *fliQ *genes which are located downstream of the *fliY *gene in the same operon. The mutant displayed visibly weakened rotative motion in liquid medium and its migration on semisolid medium was also markedly attenuated compared to the wild-type strain. Compared to the wild-type strain, the mutant showed much lower levels of adhesion to murine macrophages and apoptosis-inducing ability, and its lethality to guinea pigs was also significantly decreased.

**Conclusion:**

Inactivation of *fliY*, by the method used in this paper, clearly had polar effects on downstream genes. The phentotypes observed, including lower pathogenicity, could be a consequence of *fliY *inactivation, but also a consequence of the polar effects.

## Background

The genus *Leptospira *is composed of both saprophytic and pathogenic species [1]. Pathogenic *Leptospira *spp., such as *L. interrogans*, *L. borgpetersenii*, *L. weilii *and *L. kirschner*, are the causative agents of leptospirosis, a serious world-wide disease in humans and animals [2,3]. The disease in humans occurs mostly after contact, often through skin wounds, with soil or water contaminated by urine of infected animals. Its severity varies from mild to rapidly fatal. Severe symptoms are characterized by visible jaundice involving hepatic injury, acute renal failure, carditis and hemorrhage, and case fatality varies from a few percent to 25% [3-6]. However, the mechanisms of disease caused by pathogenic *Leptospira *spp. remain largely unknown.

Both pathogenic and saprophytic leptospires express two endoflagella (periplasmic flagella). One of the endoflagella is attached at one end of the cell and is located between the protoplasmic cylinder and the outer membrane sheath [7-9]. The endoflagella, rotating within the periplasmic space, are responsible for spirochete motility. In pathogenic *Leptospira *species, this motility is considered to contribute to invasion into hosts and diffusion within the hosts during infection [9,10]. In previous studies, we found that pathogenic leptospires can adhere to host cells with one or two termini of the microbial bodies, while non-pathogenic leptospiral strains lacked this ability [11,12]. The adhering positions were located at the terminal knobs in which flagellar basal bodies are found [1,7]. At the bottom of the flagellar structure, there is a basal body composed of MS and C rings [13,14]. In flagellated bacteria, some proteins in the Fli family form the C ring, which functions as the flagellar rotor and contains the directional switching capability of the flagellar motor [15-18]. However, a possible role for the leptospiral endoflagella in pathogenicity has never been explored.

A complete set of flagella-associated genes were found in the genomic sequences of *L. interrogans *serovar Lai strain Lai and serovar Copenhageni strain Fiocruz L1-130, including four genes that encode flagellar motor switch proteins (FliG, FliM, FliN and FliY) [19,20]. In bacteria, the flagellar motor switch proteins play a critical role in control of flagellar motor direction [14,17,18]. Thus far FliY has been found in some spirochetes and a few bacteria but does not exist in most bacteria [21,22]. Particularly, FliY of *Bacillus subtilis *was shown to be a CheY-P-hydrolyzing protein in the chemotactic signaling cascade [22]. In addition, leptospiral FliY carries a carboxy-terminal domain of 60 amino acid residues that is homologous to a domain of YscQ in *Yersinia pestis *[19,20]. The YscQ protein was identified as a member of the flagellar associated type III secretion system (T3SS), with multiple functions such as controlling the directional rotation of flagella and the export of virulence factors including Yop proteins [23,24]. The C ring of *Escherichia coli *does not have FliY, but its FliN has a high sequence homology with FliY of *L. interrogans *strain Lai [19] and FliN is an essential agent for motility and virulence protein export [25]. These data suggest that FliY of pathogenic *Leptospira *species may have important functions in motility and virulence.

In the present study, we constructed a *fliY *gene knock-out (*fliY*^-^) mutant of *L. interrogans *serovar Lai strain Lai based on homologous recombination using a suicide plasmid. To examine the possible role of FliY in pathogenesis, the mutant and wild-type strain were compared in assays of motility in liquid medium and migration on semisolid agar, adhesion to macrophages, stimulation of apoptosis in infected host cells, and lethality to guinea pigs.

## Results

### Products of *fliY *gene amplification and rFliY expression

**Figure 1:**
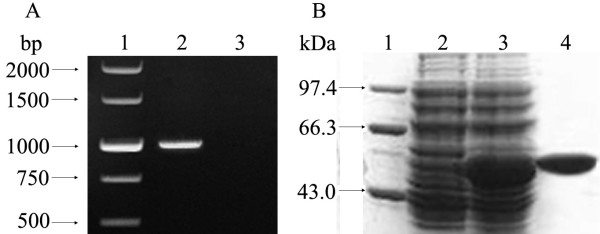
**Amplification and expression of the *fliY *gene and purification of the rFliY protein**. Panel A, showing PCR analysis. Lane 1: DNA marker (TaKaRa, China); lane 2: the amplification segment of the entire *fliY *gene; lane 3: blank control. Panel B, showing SDS-PAGE analysis. Lane 1: protein marker (TaKaRa); lane 2: pET32a with no insertion of the *fliY *gene; lane 3: the expressed recombinant protein, rFliY; lane 4: the purified rFliY protein.

The amplification segments with expected size of the entire *fliY *gene (1065 bp) from *L. interrogans *serovar Lai strain Lai were obtained by PCR (Fig 1A). The cloned *fliY *gene had 100% nucleotide sequence identity with the reported sequences in GenBank (Accession No.: NC_004343, NC_005823) [10,11]. The recombinant plasmid, *E. coli *BL21DE3^pET32a-*fliY*^, expressed rFliY under inducement of isopropyl-β-D-thiogalactopyranoside (IPTG), and the purified rFliY by Ni-NTA affinity chromatography showed a single band on a polyacrylamide gel after electrophoresis (Fig 1B). The rabbits immunized with rFliY could produce rFliY-specific serum antibody and the immunodiffusion titer of antiserum against rFliY was 1:4.

### Characterization of the *fliY*^- ^mutant

**Figure 2:**
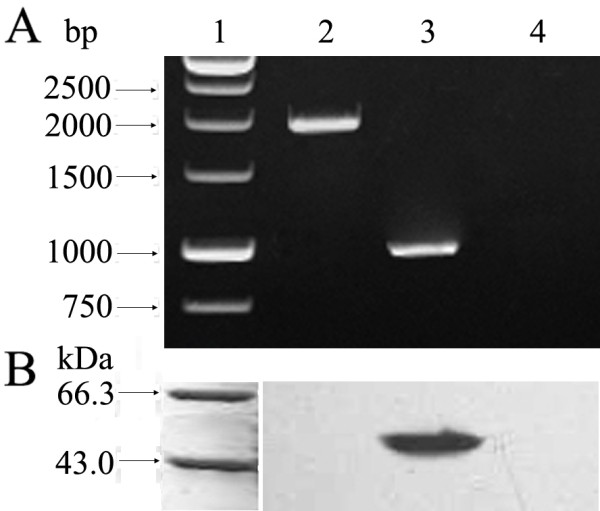
**Confirmation for insertion mutantion of *fliY *gene in the *fliY*^- ^mutant**. Panel A, showing PCR analysis. Lane 1: DNA marker (TaKaRa); lane 2: the amplification segment (2019 bp) of mutated *fliY *gene from the *fliY*^- ^mutant; lane 3: the amplification segment (1065 bp) of the *fliY *gene from the wild-type strain; lane 4: blank control for PCR. Panel B, showing Western Blot analysis. Lane 1: protein marker (TaKaRa); lane 2: the *fliY*^- ^mutant lacking the FliY protein; lane 3: the wild-type strain expressing the FliY protein; lane 4: blank control for Western Blot assay. rFliY antiserum was used as the primary antibody.

**Figure 3:**
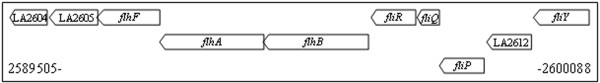
**Genes present with the *fliY *gene within the same predicted operon**. Annotation of the genes (gene name/product): *fliY*/flagellar motor switch protein; LA2612/flagellar protein required for flagellar formation; *fliP*/flagellar biosynthesis protein; *fliQ *and *fliR*/flagellar biosynthetic proteins and type III secretion apparatus proteins; *flhB*/flagellar protein; *flhA*/flagellar biosynthesis protein; *flhF*/flagellar GTP-binding protein; LA2605/ParA protein; and LA2604/hypothetical protein.

To create a *fliY*^- ^mutant of *L. interrogans*, we cloned the *fliY *gene into p2NIL and inserted an ampicillin gene at the *Bgl *II site near the 5' end. This plasmid was then introduced into *L. interrogans *followed by selection for ampicillin resistance, to create a *fliY bla *mutant. Sequencing data indicated that the *fliY *gene and ampicillin resistance gene (*bla*) segments in suicide plasmid p2NIL^*fliY*-*bla *^had the same orientation, and the nucleotide sequences were the same as in the original cloned *fliY *and *bla *genes. The *fliY*^- ^mutant could grow in 100 μg/ml ampicillin-contained Korthof liquid medium for at least 3 months in our laboratory. The generation time of the mutant (about 10 d) was the same as that of the wild-type strain. Subsequent PCR analysis confirmed that the mutant maintained a modified *fliY *gene that was larger (2019 bp) than the wild-type gene (1065 bp), into which inserted the ampicillin resistance gene (954 bp) had been inserted (Fig 2A). The Western Blot analysis also revealed the absence of expression of FliY in the mutant (Fig 2B). Furthermore, the absence of mRNAs for the *fliP *and *fliQ *genes, downstream of *fliY *gene, indicated that the transcription of the two genes were inhibited (data not shown). In fact, ten genes (*fliY*, LA2612, *fliP*, *fliQ*, *fliR*, *flhB2*, *flhA*, *flhF*, LA2605 and LA2604) should be transcribed by the same operon, based on the genome structure predicted by the software, MicrobesOnline Operon Predictions (Fig 3).

### Persistently lower motility of the *fliY*^- ^mutant

**Figure 4:**
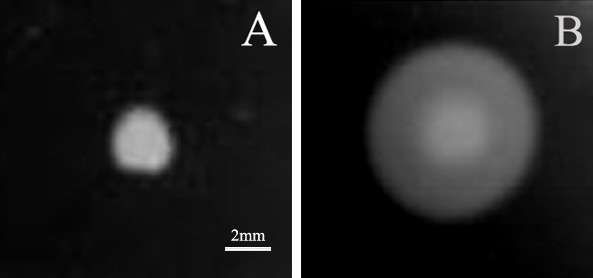
**Colony sizes of the *fliY*^-^mutant and wild-type strain on semisolid Korthof agar**. The colonies with different sizes formed by the *fliY*^- ^mutant (A) and wild-type strain (B) on semisolid Korthof agar. The leptospires were cultured on 8% RS semisolid Korthof plate for three weeks. This experiment was repeated three times.

Normally, leptospires have a typical motive manner with rotation. However, all microbes of the *fliY*^- ^mutant in liquid Korthof medium by dark-field microscopy only had 40% of rotative motion frequency per minute of the wild-type strain, but presented a similar shape to the wild-type strain (data not shown). On semisolid Korthof agar plates, the colonies of the *fliY*^- ^mutant were noticeably smaller (2-3 mm in diameter) than that of the wild-type strain (6-8 mm in diameter) (Fig 4), consistent with attenuated motility of the mutant.

### Altered adhesion of the *fliY*^- ^mutant

**Figure 5:**
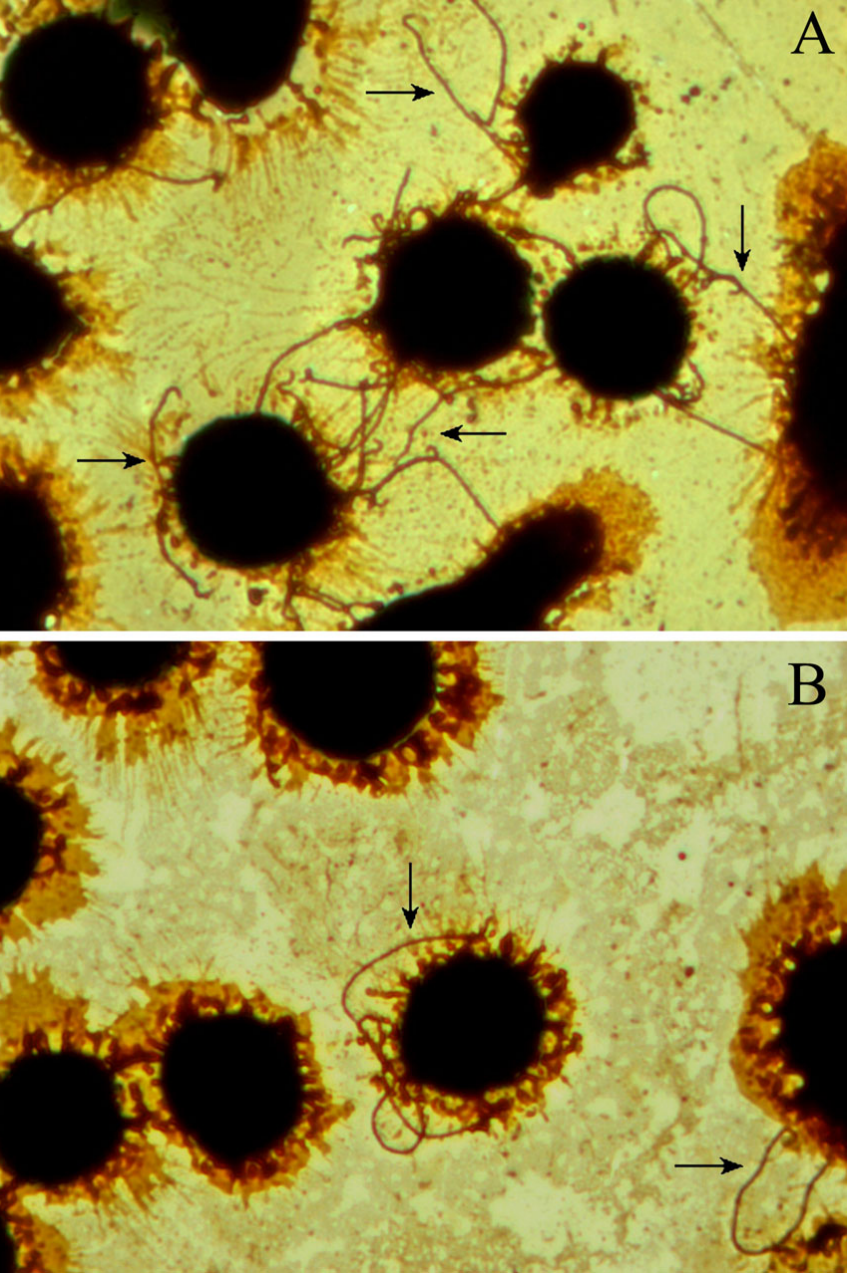
**Adhesion of the *fliY*^- ^mutant and wild-type strain to J774A.1 cells**. Adhesion of the wild-type strain (A) and *fliY*^- ^mutant (B). The arrow indicates the adhering leptospires on J774A.1 cells. This experiment was repeated three times. Magnification × 400.

**Figure 6:**
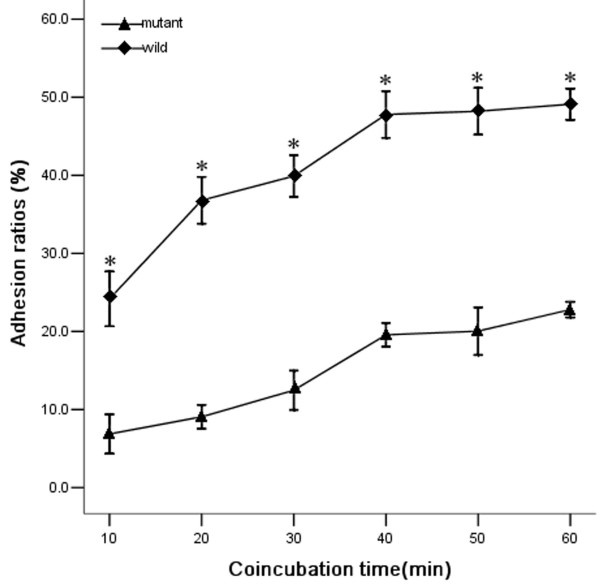
**Adhesion ratios of the fliY^- ^mutant and wild-type strain to J774A.1 cells after different incubation times**. Adhesion was quantified as described in Methods. *: *P *< 0.05, wild-type strain compared with the mutant.

The wild-type *L. interrogans *strain Lai could adhere to the surface of J774A.1 cells with one or both bacterial ends (Fig 5A). The attached wild-type leptospires were visible on the cell surface after 10 min post inoculation (p.i.) and the adhesion ratios approached a plateau after 40 to 60 min p.i. (Fig 6). However, the *fliY*^- ^mutant was significantly impaired in its ability to adhere to the macrophages, compared to the wild-type strain (*P *< 0.05) (Fig 5B and Fig. 6).

### Host-cell apoptosis induced by the wild-type and the *fliY*^- ^mutant strains

**Figure 7:**
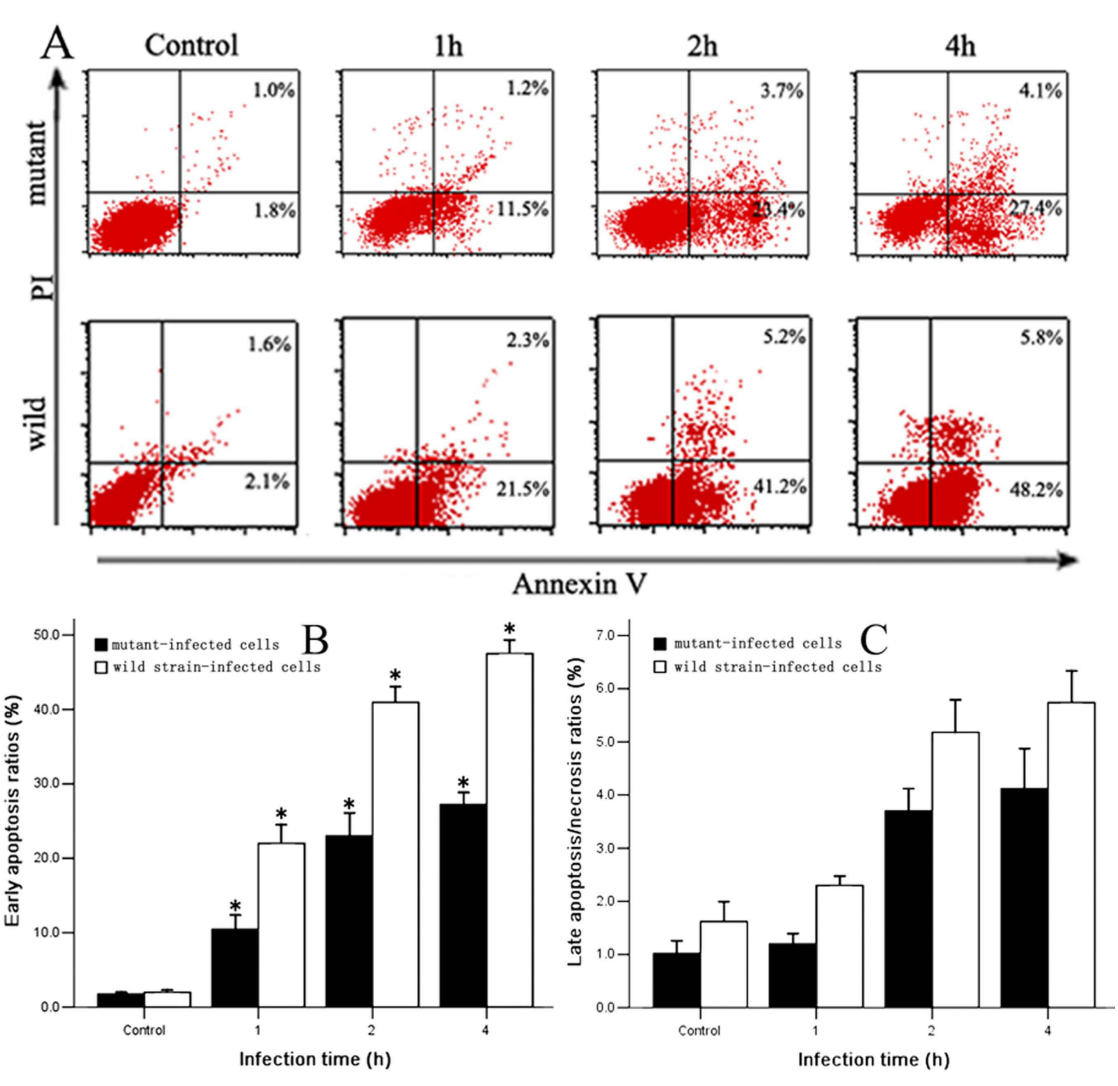
**Apoptosis ratios of J774A.1 cells induced by the *fliY*^- ^mutant and wild-type strain**. Panel A: lower left quadrants indicate unstained normal cells; lower right quadrants, the early apoptotic cells binding Annexin-V; upper left quadrants, the necrotic cells binding PI; and upper right quadrants, the late apoptotic/necrotic cells binding both Annexin-V and PI. Cell death was measured by flow cytometry as described in Methods. Panel B: proportion of early apoptotic cells (annexin-V^+^/PI^-^) after infection for different times. The data are expressed as mean ± SD for three independent experiments. Panel C: proportion of late apoptotic/necrotic cells (annexin-V^+^/PI^+^) after infection for different times. The data are expressed as mean ± SD for three independent experiments. *:*P *< 0.05, wild-type strain compared with the mutant.

As shown in Fig 6, the wild-type *L. interrogans *strain Lai induced apoptosis of J774A.1 cells, and the maximal apoptotic ratio (48.2 ± 2.9%) appeared after 4 h coincubation, as detected by flow cytometry (Fig 7A). However, the ability of the *fliY*^- ^mutant to cause apoptosis was markedly decreased, and the levels of apoptosis and late apoptosis/necrosis at all the different incubation times were significantly lower than those induced by the wild-type strain (*P *< 0.05) (Fig 7B and 7C).

### Attenuated lethality of the *fliY*^- ^mutant strain in guinea pigs

The lethality to guinea pigs of the wild-type *L. interrogans *strain Lai was significantly larger than of the *fliY*^- ^mutant during a 10d post-challenge period (Table 1). No animals infected by the *fliY*^- ^mutant strain died comparing with 100% death, which were infected by wild-type strain with the same dosage. When the challenge dosage for the *fliY*^- ^mutant was increased to ten times the dosage used for the wild-type strain, only 60% of the animals infected with the *fliY*^- ^mutant died.

**Table 1:** Lethality of the *fliY*^- ^mutant and the wild-type strain in infected guinea pigs.

Strain	Challenge dosage (×10^8 ^per animal)	Animal (n)	Dead/surviving (n/n)	Death rate (%)
Wild-type Mutant	6	10	10/0	100
	6	10	0/10	0
	12	10	0/10	0
	30	10	0/10	0
	60	10	6/4	60

## Discussion

Recent reports have shown that flagellin and other flagella-associated proteins from many bacteria participate in adhesion to host cells and colonization of hosts [26-28]. In vitro studies have suggested that the role of flagella could be to increase invasion into host cells and survival within macrophages [29,30]. However, the correlation between flagella and pathogenicity of pathogenic *Leptospira *spp. had not been investigated until now. *L. interrogans *serogroup Icterohaemorrhagiae serovar Lai strain Lai is the most prevalent pathogenic leptospiral strain, which is responsible for over 70% of human leptospirosis cases in China [31]. We therefore inactivated the *fliY *gene in *L. interrogans *strain Lai using a suicide plasmid, which is a frequently adopted strategy for determining the function of a target gene. Recently, Croda and his colleagues used plasmid pB2SK to successfully construct a suicide plasmid with spectinomycin resistance for inactivating the *ligB *gene of *L. interrogans *serovar Copenhageni strain Fiocruz L1-130 [32]. In the present study we first used another plasmid, p2NIL, with an ampicillin resistance gene (*bla*) to construct a *fliY *gene knock out (*fliY*^-^) mutant. A *fliY*^- ^mutant has been constructed, but that *fliY *inactivation by ampicillin cassette insertion also negatively affected downstream genes; therefore, care has to be taken when interpreting the phenotypes observed for this mutant.

The inactivation of the *fliY *gene has shown different effects on formation of flagella in different bacteria. In *Bacillus subtilis*, the deletion of *fliY *resulted in the loss of flagella [33]. However, the flagella were still produced in the *fliY*-deleted strain of *Bacillus cereus *[34]. Although the leptospiral *fliY*^- ^mutant generated in this study displayed remarkably attenuated motility compared to the wild-type strain, it maintained the typical spiral shape and propeller movement which is caused by the periplasmic endoflagella [1,7]. As mentioned previously, the major function of flagellar motor switch proteins is to control flagellar motor direction [16,19-22]. Thus, we infer that the *fliY *gene inactivation should not affect the formation of the endoflagella.

It is well known that adhesion to host cells is a primary and critical step for bacterial infection [35,36]. Recently, the importance of cell adhesion for pathogenic *Leptospira *spp. has been demonstrated [11,12,37,38]. Adhesion to host cells also acts as an essential role for pathogenicity of other spirochetes [39,40]. Mononuclear macrophages are the most important phagocytes in the human innate and acquired immnune systems. However, many pathogenic bacteria can evade host immunity by inducing apoptosis of macrophages [41-43]. Similarly, pathogenic *Leptospira *spp. can escape from the host immune system by promoting macrophage apoptosis [11,44-46]. In the present study, we provide evidence that the ability of the *fliY*^- ^mutant to adhere to J774A.1 cells, to induce apoptosis in the cells, and to cause death in guinea pigs is much lower than for the wild-type strain. All the phentotypes observed, including lower pathogenicity, could be a consequence of *fliY *inactivation, or a consequence of the polar effects, or of both.

T3SS is one of protein export systems used by most Gram-negative bacteria [47]. Morphologically, as a transmembrane channel, T3SS is composed of multiple protein complexes called an injectisome, responsible for transporting virulence factors into host cells, some of which cause cell metabolic disorder and death [47-49]. However, the flagellar export apparatus can also function as a bacterial virulence protein secretion system [50]. For example, FliF of *Pseudomonas aeruginosa*, a flagellar associated protein component in the MS ring, is involved in adhesion by controlling secretion of bacterial adhesins [51]. Although the T3SS and flagellar export apparatus are two relatively separate systems in many pathogenic bacteria [52], the T3SS and flagellar export apparatus in *Yersinia enterocolitica *play a common role in secretion of bacterial phospholipases during infection [53]. Taken together, these observations suggest that inactivation of the leptospiral *fliY *gene (or of the downstream located *fliPQ *genes) may decrease the export of some unknown adhesion- and cytotoxicity-associated virulence proteins.

## Conclusion

Inactivation of *fliY *clearly had polar effects on downstream genes. The phentotypes observed, including decreasing motility, adhesion to macrophages and host-cell apoptosis, and attenuating lethality in infected guinea pigs, could be a consequence of *fliY *inactivation, but also a consequence of the polar effects.

## Methods

### Bacterial strains and cell lines

*L. interrogans *serogroup Icterohaemorrhagiae serovar Lai strain Lai was offered by the National Institute for the Control of Pharmaceutical and Biological Products in Beijing, China. The leptospires were cultured in Korthof liquid medium containing 8% heat-inactivated rabbit serum (RS) at 28°C. To maintain virulence, the strain was passaged intraperitoneally in specific pathogen-free Dunkin-Hartley ICO:DH (Poc) guinea pigs (2 weeks old, each weighing about 120 g) before use, according to the description by Merien et al. and Viriyakosol et al. [44,54]. Animal protocols were approved by the Animal Ethics Review Committee of Zhejiang University.

### Cell line and culture

The murine mononuclear-macrophage-like cell line (J774A.1) was obtained from the American Type Culture Collection (Rockville, MD, USA). The cells were cultured in RPMI 1640 medium (GIBCO, USA), supplemented with 10% heat-inactivated fetal calf serum (FCS) (GIBCO), 100 U/ml penicillin and 100 μg/ml streptomycin (Sigma, USA) at 37°C in an atmosphere of 5% CO_2_.

### PCR and sequencing

Genomic DNA of *L. interrogans *strain Lai was extracted using Bacterial Genomic DNA Extraction Kit (BioColor, China). Plasmid pUC19, which has an ampicillin resistant gene (*bla*) cassette including promotor in *E. coli *DH5a, was prepared by Mini-plasmid Rapid Isolation Kit (BioDev, China). Primers for amplifications of the *fliY *and *bla *genes are shown in Table 2. A commercial PCR Kit (TaKaRa, China) was used to amplify the *fliY *and *bla *genes. The products were detected on 1.5% ethidium bromide pre-stained agarose gel by electrophoresis, purified using PCR Product Purification Kit (BioColor), and ligated into plasmid pUCm-T using T-A Cloning Kit (BioColor) to form recombinant plasmids pUCm-T^*fliY*^. pUCm-T^*bla *^sequencing was performed by Invitrogen Co. Ltd in China.

**Table 2:** Primer information for amplification of the *fliY *and *bla *genes.

Gene	Primer sequence (5'-3')	Product size
*fliY*	F: GCC GGA TCC (*Bam*H I) ATG GGT GAA GGT TCC CTA TCA CAG	1065 bp
	R: GCC AAG CTT (*Hind *III) TCA CTT ACC CTC CGG CTT AAT CCG	
*bla*	F: GCC AGA TCT (*Bgl *II) TCT AAA TAC ATT CAA ATA TGT	954 bp
	R: GCC AGA TCT (*Bgl *II) CTT GGT CTG ACA GTT ACC AAT	
*fliP*	F: ATG AAA ATG AGA CAT AAA	804 bp
	R: TCA TTT ATA ACT CCT TAC	
*fliQ*	F: ATG ACG GAA TTA GAC GTT ATG	264 bp
	R: CTA AAA TTT TTC GAT CAT CAA	

F: forward primer, R: reverse primer.

### Expression, purification and immunization of recombinant FliY

pUCm-T^*fliY *^and expression vector pET32a (Novagen, USA) were digested with *Bam*H I and *Hind *III, respectively. The recovered *fliY *segment was ligated into linearized pET32a using T4 DNA ligase (TaKaRa), and then transformed into *E. coli *BL21DE3 (Novagen) to form *E. coli *BL21DE3^pET32a-*fliY*^. Recombinant FliY (rFliY) was expressed under inducement of 0.5 mM IPTG for 4 h at 37°C. The expressed rFliY was extracted by Ni-NTA affinity chromatography and the purity of rFliY was determined by SDS-PAGE. New Zealand rabbits, provided by the Laboratory Animal Center of Zhejiang University, were immunized intradermally four times at an interval of once a week with the purified rFliY that was pre-mixed with Freund's adjuvant. On the 15th day after the last immunization, the rabbit serum was collected and the immunodiffusion test was used to examine the titer of antiserum.

### Generation and characterization of the *fliY*^- ^mutant

**Figure 8:**
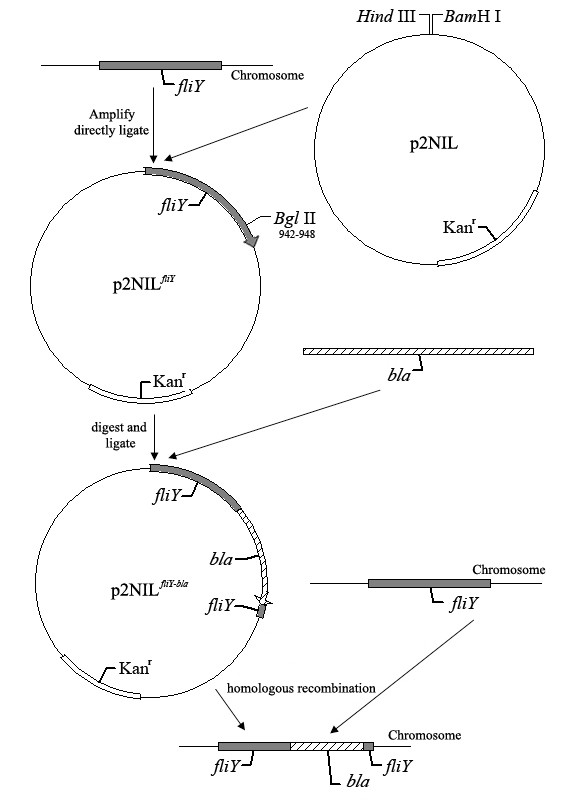
**Strategy for preparing the *fliY*^- ^mutant using the suicide plasmid p2NIL^*fliY*-*bla*^**.

Plasmid p2NIL used in this study was kindly offered by Dr. Tanya Parish and Dr. Amanda C. Brown. The *fliY *segment from pUCm-T^*fliY *^was inserted into p2NIL at the *Bam*H I/*Hind *III sites to form p2NIL^*fliY*^. The plasmid has an origin of replication for *E. coli (oriE)*, a kanamycin resistance gene (*kan*), and a multiple cloning site [55]. Since there is a unique *Bgl *II site within the *fliY *gene sequence (942th-947th bp at the 5' end), p2NIL^*fliY *^was cut with *Bgl *II, dephosphorylated and ligated with ampicillin amplification segment (*bla*) including the promotor (10th-16th bp at 5' end) flanked by a *Bgl *II site to form a suicide plasmid, p2NIL^*fliY*-*bla*^. The suicide plasmid was transformed into *E. coli *DH5a for amplification in Luria-Bertani (LB) medium supplemented with both 100 μg/ml ampicillin and 50 μg/ml kanamycin, and then recovered for sequencing. The p2NIL^*fliY*-*bla *^plasmid was then denatured by alkali treatment as previously described [56,57], and electrocompetent leptospires were prepared according to Saint Girons' protocol [58]. The competent leptospiral cells were mixed with 2 μg p2NIL^*fliY*-*amp *^DNA, and then bathed on ice for 10 min for electrotransformation. Finally, the mixture was transferred to 1 ml of 8% RS Korthof liquid medium for a 48 h incubation at 28°C. The *fliY*^- ^mutant was selected on 8% RS Korthof plates containing 100 μg/ml ampicillin. Individual ampicillin-resistant colonies were inoculated in 8% RS Korthof liquid medium supplemented with 100 μg/ml ampicillin. The steps to construct the suicide plasmid and to generate *fliY*^- ^mutant are summarized in Fig 8.

### Confirmation of the *fliY *gene inactivation in mutants

The *fliY*^- ^mutant was cultured at 28°C in 8% RS Korthof liquid medium containing 100 μg/ml ampicillin. Genomic DNA of the mutant was extracted using Bacterial Genomic DNA Extraction Kit (BioColor), and the disrupted *fliY *gene in the mutant was identified by PCR and the Western Blot assay. The product of the *fliY-bla *gene is larger in the mutant (2019 bp) than the *fliY *gene in the wild-type strain (1065 bp). By using 1:2500 diluted anti-rFliY serum as the primary antibody and 1:3000 diluted HRP-labeling goat anti-rabbit IgG (Jackson ImmunoResearch Laboratories, USA) as the secondary antibody, a Western Blot assay was performed to detect the expression of FliY protein in the mutant. In the genomic sequence of *L. interrogans *serovar Lai strain Lai, the *fliP *and *fliQ *genes are located downstream from the *fliY *gene. In order to further confirm the inactivation of the *fliY *gene, two separate RT-PCRs were performed to detect mRNAs of the *fliP *and *fliQ *genes, with primers shown in table 2. In addition, an operon predictor tool http://www.microbesonline.org/ was used for analysis of the operon structure.

### Motility assay

The motility and shapes of the *fliY*^- ^mutant and wild-type strain in 8% RS Korthof liquid medium were observed under dark-field microscope after incubation at 28°C for 10 d (the primary generation), 50 d (the 5th generation) and 100 d (the 10th generation). The colony sizes of the mutant and wild-type strain on 8% RS semisolid Korthof plate (0.25% agar) that had been incubated at 28°C for three weeks were measured for three times as described above.

### Fontana silver staining

J774A.1 cells (5 × 10^4 ^cells/ml) were seeded on coverslips in 12-well tissue culture plates (Corning, USA) and pre-incubated for 24 h at 37°C in an atmosphere of 5% CO_2_. The freshly cultured leptospires of the *fliY*^- ^mutant and wild-type strain were harvested by centrifugation (12,000 × g, 15min, 15°C) and washed twice with autoclaved PBS. The pellets were suspended in pre-warmed antibiotics-free 10% FCS RPM1640 to a final concentration of 10^8 ^leptospires/ml by dark-field microscopy with a Petroff-Hausser counting chamber (Fisher Scientifics, PA). The cell monolayers were washed three times with autoclaved PBS and then infected with each of the suspensions at an MOI of 100 (100 leptospires per cell) for 10, 20, 30, 40, 50 and 60 min, respectively. After infection, the coverslips were washed three times with PBS to remove non-adherent leptospires, fixed in 5% formaldehyde, stained with silver nitrate, and observed under a light microscope [59]. The adhesion ratio was defined as the number of adhering leptospires per 100 infected host-cells × 100% [11].

### Assessment of cell death by flow cytometry

Apoptosis was measured by flow cytometry using annexin-V-fluorescein isothiocyanate (FITC)/propidium iodide (PI) labeling as previously published [11,60]. The J774A.1 cell monolayers were infected with either the *fliY*^- ^mutant or wild-type strain with an MOI of 100 at 37°C for 1, 2, or 4 h [46]. After infection, the cells were washed three times with PBS, collected with a cell scratcher, and centrifuged at 1,000 × g for 5 min. The pellets were washed three times with PBS, resuspended in annexin-V binding buffer with FITC-conjugated annexin-V, and incubated for 15 min at room temperature in the dark, following the manufacturer's instructions (Caltag Laboratories, USA). After PI was added, the cell suspension was immediately analyzed by FACSCalibur flow cytometry and CellQuest Pro software (Beckman Coulter, USA). Cells in the early apoptotic phase bind annexin-V but exclude PI, and those in the late apoptotic/necrotic phase stain with both annexin-V and PI, while necrotic cells stain with PI alone [60].

### Animals and challenge infections

The Dunkin-Hartley guinea pigs (150 ± 5 g, 3 weeks old) used in this study were provided by the Laboratory Animal Center of Zhejiang University. The animals were challenged intraperitoneally with different dosages of either wild-type *L. interrogans *serovar Lai strain Lai or the *fliY*^- ^mutant, and then observed for 10 d [1]. The animal experiments were approved by the Animal Ethics Review Committee of Zhejiang University.

### Statistical analysis

Data from a minimum of three experiments were averaged and presented as mean ± SD (standard deviation). One-way analysis of variance (ANOVA) followed by the Dunnett's multiple comparisons test were used to determine significant differences. Statistical significance was defined as P value ≤ 0.05.

## Authors' contributions

SL carried out the molecular genetic studies, immunoassays and drafted the manuscript. AS cultured the leptospires and participated in immunoassays. DMO participated in study design and revised the manuscript. SW and JZ carried out analysis and interpretation of data. JY conceived of the study, and participated in its design and coordination. All authors read and approved the final manuscript, and agreed to having it published.
